# “Not Today, Diabetes”: Using Blog Analysis to Understand Emotional Interactions and Support Among People With Type 1 Diabetes

**DOI:** 10.3389/fcdhc.2020.613569

**Published:** 2021-01-06

**Authors:** Heather L. Stuckey, Sean M. Oser, Erin L. Miller, Tamara K. Oser, Mark Peyrot, Aditi Sharma

**Affiliations:** ^1^ Department of Medicine, College of Medicine, Pennsylvania State University, Hershey, PA, United States; ^2^ Department of Family Medicine, School of Medicine, University of Colorado Anschutz Medical Campus, Aurora, CO, United States; ^3^ Department of Family Medicine, College of Medicine, Pennsylvania State University, Hershey, PA, United States; ^4^ School of Medicine, Loyola University Maryland, Baltimore, MD, United States; ^5^ Department of Public Health Sciences, College of Medicine, Pennsylvania State University, Hershey, PA, United States

**Keywords:** blog, emotional support, qualitative research, type 1 diabetes, diabetes online community

## Abstract

The goal of this study is to understand how internet blogs are used by people with type 1 diabetes (T1D) to provide or exchange social support. A stratified, clustered proportionate probability sample of entries from 10 Internet blogs focusing on T1D was obtained. A random sample of 100 days generated 200 blogger posts and 1,606 commenter responses. Entries were coded using qualitative analysis software and analyzed thematically. Blogs were used as a dynamic, interactional form of emotional support from others who understood diabetes from personal experience; and as a source of sharing lived user experience of having diabetes, more often than as a way of communicating medical knowledge or facts about diabetes. Blog participation contributed to a sense of belonging for participants in the “Diabetes Online Community” where there was a shared culture. In conclusion, blogs provide unobtrusive access to the experiences of people with T1D that are driven by their interests rather than those of qualitative research interviewers or healthcare providers. In addition to permitting analysis of the way that participants use blogs to address their own personal wants and needs, blog data can serve as an inexpensive and unobtrusive method for studying topics of interests to researchers and healthcare providers.

## Introduction

Few health conditions require as much self-management as type 1 diabetes (T1D) (1, 2). Due to the complexity of the condition and its management, people with T1D (PWT1D) require various types of support for self-management and social/emotional support (3–7). Traditional sources of support have been healthcare providers (HCPs), support groups, family members and friends. The Internet has emerged as a wealth of ongoing, interactive information and social support for diabetes (8–14). Illness blogs (online journals about a content area) allow for study of the experience of illness in a naturalistic and longitudinal manner, often with greater detail than data relying only on participant recall. Participants produce online illness blogs to share their own illness narratives and connect with others going through similar processes (15). Social media has enabled PWT1D to find and maintain connections with peers with T1D for self-management support. “Diabetes Online Community” (DOC) is a widely used term that encompasses all of the people who engage in various online activities related to living with diabetes across a collection of web-based platforms including community forums, blogs and social media sites such as Facebook and Twitter (16).

The theory of social constructivism suggests that as people share background knowledge and participate in collaborative activities, they negotiate meaning and build knowledge, not as individuals, but as a community (17). An online community provides opportunity for expression to make sense of the condition through sharing, negotiating, and building knowledge. As such, social media can increase the sense of connectedness (18), and the use of social media has been increasing as an area of research interest related to T1D self-management and support (10, 19–22).

One form of social media is blogs, which provide insights, information, and comments on a specific topic area. In recent years, blogging activity has increased dramatically—from just 4% of social media usage in 2008 to 47% in 2018 with 3.196 billion social media users worldwide (23–25). Unlike other social networking forums, such as Facebook and Twitter, blogs provide sustained and focused dialogue with peers, remain accessible and open to all who seek or subscribe to them, are socially constructed, and are often moderated by peers who filter inaccurate or harmful comments and commercial promotions (26). Moderated discussions have been found to result in increased social communication, improved participation, and increased trust among participants (27).

Blogs are one way that the people of the DOC create and share culture together in a virtual community, comprised of three roles: (a) “Bloggers” journal their experiences for others; (b) “Commenters” read and actively comment on others’ posts, and they may or may not also be bloggers themselves; and (c) “Lurkers” read others’ blogs without commenting (28). With approximately 85% of the estimated 1.25M Americans with T1D being adults (and 3M people worldwide), and with 40,000 new T1D diagnoses annually (29), it is likely that blogs will continue to be a significant source of information and self-management support for PWT1D (30). Popular T1D blogs, such as *SixUntilMe, Scott’sDiabetes*, and *DiabetesMine*, are viewed approximately 90,000 times per month, with 45,000 of these views by unique individuals. Blogging activity has positive association with health outcomes and is valuable as a means of providing support and promoting self-management (14, 16, 31).

One venue for dissemination of insights from and benefits of T1D blogs is through citations and referrals by healthcare providers (HCPs). However, many HCPs are not engaged with the same types of social media as their patients (30) and therefore are not presently serving as collaborators on T1D blogs to inform and provide guidance (32). The Association of Diabetes Care & Education Specialists (ADCES) has endorsed referral to the DOC and has developed a handout to provide to PWT1D to refer them to the DOC (Warshaw & Edelman, 2019), however only one in 3 diabetes educators (34.7%) recommends the DOC to their patients (33). Recently, the ADCES further endorsed referral to online peer support communities such as the DOC in their 2019 Perspectives in Practice (34).

Although much has been written about the complexities of managing T1D, some HCPs do not understand the day-to-day realities of living with such a complex condition (35). Studies show discordance between PWT1D and HCP perceptions of different barriers to diabetes care and their importance (36, 37). Whereas HCPs perceived informational barriers as most important and believed that people with diabetes (PWD) wanted more education, PWD wanted more psychosocial support and identified psychological barriers as most important (6). PWD may be able to get some of this support from their HCPs, but PWD may know more about certain aspects of life with diabetes and its self-management than do their HCPs (37), and they may turn to peers for such support and to tackle these daily life self-management issues (38–40).

In this paper we examine the use of blogs to understand how the DOC provide and exchange support to one another.

## Materials and Methods

The Internet ethnography (“netnography”) (41) methods used in this study were approved by the Penn State College of Medicine Institutional Review Board.

### Recruitment and Sampling Strategy

Through snowball sampling (42), ten online blogs written by adults with T1D were selected for analysis (list included in **Table 1**). Sampling began with the most visited and visible adult T1D blog, *Six Until Me* (www.sixuntilme.com). Identified blog authors gave permission to retrospectively analyze data publicly available on their blogs posted between June 1, 2012 and July 31, 2014.

**Table 1:** Blogs included in study.

Blog Name	Posts	Number of Total Comments	Number of Unique Commenters
Bittersweet-Diabetes	21	154	71
Diabetesaliciousness	28	102	95
InDpendence	15	43	21
Ninjabetic	14	60	46
The Perfect D	17	110	53
Scott’s Diabetes	12	96	59
Six Until Me	44	654	255
Strangely Diabetic	11	0	0
Sweetly Voiced	9	89	54
Texting My Pancreas	29	298	141
Six Until Me (2019)	59	472	274
Diabetesaliciousness (2019)	22	34	10
**Total**	**281**	**2112**	**1079**

Blog posts including comments were selected for inclusion based on a stratified, clustered, proportionate probability sampling strategy in which calendar days were randomly selected. Any blog posts on the randomly selected date were included in the data set, representing a cluster of content. This ensured a probability sample of all content from the ten blog sites, as blogs published more frequently had a higher likelihood of having published on any given date, and therefore a higher proportion of content included in the sample.

### Data Analysis

Data were imported into a qualitative data management program (Nvivo 10). The primary coders (ELM, TKO) reviewed the data and made memos of initial codes to form a coding scheme (primary codes and sub-codes), which was revised by the study team. To assess inter-coder reliability, the primary coders coded 10% of the data (kappa = 0.97). Coding proceeded past saturation for an additional 20% of the dataset to confirm saturation. After coding, data were processed by “sifting”, which involved identifying and selecting the most essential data from the coded items (43). The first stage of sifting sorted the data (codes) into two categories: one that related to the research question of emotional and practical support shared among T1D blog participants; and one that did not. The resulting blog data from the first stage were ‘sifted,’ into two primary analytic themes where blogs were used as a: (a) dynamic, interactional form of emotional support from others who understood diabetes from personal experience; and (b) source of sharing lived user experience of having diabetes, rather than as a source of communicating medical knowledge or facts about diabetes. Therefore, the thematic analysis describes how these blogs were used by PWT1D to exchange support.

In order to ensure that the data from 2014 was relevant to the present time, another round of retrospective analysis was conducted between January 2019 and December 2019. Two active blog sites were chosen for the analysis: *Six Until Me* and *Diabetesaliciousness*. The year 2019 was chosen as it is the most recent complete year. All blog posts from the two sites were analyzed.

## Results

There were approximately 2,470 blog posts within the initial 24-month sampling frame. The study team analyzed data for the first 50 randomly selected dates, and continually assessed for saturation as coding proceeded. After coding the initial 140 blog posts from the first 50 dates, saturation had not been achieved; therefore, additional randomly selected dates were analyzed. Twenty-seven (27) blog posts were excluded from analysis as irrelevant to the research question (e.g., not discussing diabetes, consisting only of a link to another site without original content, etc.). A total of 200 blog posts including 8 guest posts were included for analysis in the final sample of the 2014 data. These included variable numbers of comments, averaging 8.2 comments per blog post (range 0–38). Considering the age of the data, an additional 80 blog entries including 2 guest posts were included from 2019. Findings remain constant among the two samples.

**Table 2** lists the primary codes and sub-codes used in the thematic analysis, along with the number of occurrences and an illustrative quote.

**Table 2:** Codes used for thematic analysis.

Primary Codes	Secondary Codes, # of Occurrences, and Illustrative Quotes (in addition to quotes in the primary manuscript)
**Shared ways of coping (570)**	**Humor and sarcasm (130)** *If we can laugh at something, we can own it, and that includes diabetes.* *I often find myself in a totally silly and very entertaining conversation that leaves me giggling long after I’ve shut down the computer.*
	**T1D to T1D emotional support from DOC (363)** *Just by reading a blog post … the DOC automatically lifts my spirits, makes me smile and makes diabetes in all dimensions less challenging. And those some days become better days - And that’s so awesome!* *On the days when diabetes gets me down - It’s the [DOC] that pulls me up - Through blog posts, Instagram pictures, tweets, Facebook messages, texts or phone calls - And I am very grateful indeed~*
	**T1D to T1D informational support from DOC (41)** *It’s times like these that I’m so glad to have found the DOC. Because posts from blogs and Facebook began to flash through my mind. Someone used ketone sticks to test soda for sugar … Someone else tested soda on their meter.* *I finally learned that I use 10% less insulin in the first two weeks of my menstrual cycle than I do in the second two weeks. Now I have two different basal rate patterns that I switch every two weeks. It has made a big difference for whether or not I am low or high all the time.*
	**T1D to T1D instrumental support from DOC (36)** *The DOC … real people with real day to day D issues and that was the only key I was lacking in the clinical supports I already had! The DOC has been a huge resource for me.* *I downloaded a copy of the Diabetes Health Magazine, it has coupons for glucose tabs and other supplies, and some recipes. It’s definitely worth checking out.*
	**Affirmations (598)** *Thank you for your honesty, Karen. This was probably the most touching post I’ve read today. Hugs to you!!* *I am glad you punched through that writer’s block; this was a great post!! rage on, sometimes it’s all we can do.*
**Shared user barriers to technology (222)**	**Impact of wearing technology and carrying it with you (184)** *The pump was snatched out of my hand last night when the infusion tubing snagged on a moving ceiling fan, was whipped around at high speed and thrown violently into the closet. I would bet that nobody in the quality department at Medtronic envisioned that scenario!* *I once had a sensor on my bum, right behind the back pocket. They kept saying “ma’am, I’ll ask you one more time- please remove everything from your pockets”…the joys of diabetes.*
	**Intrusion of device alarms (17)** *Between waking up to treat lows, waking up to correct highs, the many beeps of the CGM that have disrupted my sleep, and even waking up to wonder if I’m high or low … it’s a lot of sleep I’ve been missing out on.* *I’ve taken my CGM off (for about 4 wks now) just to catch up on sleep lost from all the beep beep beeping.*
	**Lack of usability (21)** *Among my top-5 nagging annoyances with diabetes is the air-bubble-problem.* *The one issue I did have was with the touch screen - I had to touch the screen three to five times before it would register the contact.*

### Theme 1. Blogs Were Used as a Dynamic, Interactional Form of Emotional Support From Others Who Understood Diabetes in the DOC

PWT1D used words like “our” or “ours”, instead of “my” or “mine”, even though they were talking about their own personal experiences. For example, if one has to have a condition like diabetes that *“tries to control us and our happiness but fails”*, then it is best to give it a “smack in the face” by being healthy and taking care of it. The expression of personal experience was found in community:

*Why not focus on how far we’ve come in our diabetes management—And why not celebrate all the many times we’ve fallen on our “Diabetes Path”, but have gotten back up and kept on trucking!*

Advocacy regarding healthcare access and awareness regarding charities supporting T1D were constant features in the blogs:

*Access to devices that work for us matter. Access to insulin matters. Access to proper dental care and eye care matters. OUR HEALTH MATTERS, and you shouldn’t have to decide between medical care and rent.*

Emotional support and connection was an important part of the blog content. Participants affirmed each other frequently (n = 598), thanking an individual for sharing a response, or to give further encouragement.

*Karen, you are more brave [sic] than I think you know. You are not alone. Thanks for sharing, and know that you are totally worth anything it takes to feel as good about yourself as we feel about you. Which is pretty damn good.*

Instead of directing comments at an individual, bloggers and commenters in the DOC posted comments for the community-at-large to see.

*The DOC has also been a huge resource for me. It’s real people with real day to day D issues and that was the only key I was lacking in the clinical supports I already had! Thanks DOC!!!*

*Things are better for me now. I realized that I had a problem that I couldn’t fix all by myself. I sought help from counsellors, psychiatrists and doctors. I started being truthful and telling people I was struggling and couldn’t cope. I met great, wonderful friends in the DOC that, to this day, are the reason I am sitting here typing this.*

Participants believed that their medical team was helpful during medical appointments with diabetes, one even calling them “*lifesavers”*, but they did not lean on the medical team for emotional support. HCPs start by *“asking me how my diabetes was … and he wanted that answered with an A1c result”.* So PWT1D looked to the DOC for support.

PWT1D used blogs to feel like part of a community that understood diabetes from the inside out. Sometimes they used “inside” humor or sarcasm that people who did not have diabetes, or did not live with someone who had diabetes, would not find as humorous. *If we can laugh at something, we can own it, and that includes diabetes.* One example is the often unpleasant taste of glucose tablets to treat hypoglycemia:

*But hey, we want to make sure the whole DOC can voice their opinions on glucose tablet flavors. He put together a clever and funny little survey that we hope everyone will take a few seconds to answer. It contains all of the brilliant, silly and downright gross suggestions, along with spaces for your own tab flavor creations. Are you game??*

A PWT1D talked about meeting with other people who had diabetes, even though *“diabetes is no party”*, then said, *“that’s a good enough reason to put on a funny hat and celebrate”*. One person accidentally was in the wrong place at the wrong time, and a commenter said, *“I am still laughing at the mental image of an insulin pump being whipped around by a ceiling fan! That is a new one!”* to which the response was, *“Good thing I wasn’t still connected to the tubing too, right? That could have been messy”*. One person called the interactions with tubing in the restroom the *“tubing tango”*. When a stranger was looking at one PWT1D’s technology while they were waiting in line for coffee, the PWT1D *“grabbed my coffee from the counter. I smiled. And I leaned in to whisper, I am not the droid you’re looking for”*.

Some of the humor was directed at food:

*As a person with Type 1 diabetes, there is not a food on this planet that I am not permitted to consume. (There are many that you couldn’t pay me to try.)*

*I didn’t feel even a little bit bad for ordering a glass of wine on my 9 am flight. Nope.*

A hypoglycemia tip was to avoid drinking juice when wearing a white dress shirt … *“am I the only one who only buys apple juice boxes because of the lighter stain value?”*

Some issues were more serious, such as sarcasm directed toward things which cannot be immediately changed:

*Do they have a working model? Um. No. Have they figured out the anti-rejection issue? Um. No … But they do have a nifty bunch of ideas and a spiffy bunch of animations and pictures of people looking through microscopes.*

*“I’m sorry, which type of diabetes is the simple type?” I blurted out. “Because I think we would all like to sign up for that one”. I got a few laughs, but seriously … A treatment plan may be simple from a clinician’s standpoint, but I guarantee you, to the patient, their disease is challenging and frustrating, whether they are asked to make lifestyle changes and take a pill or whether they are asked to struggle with variable basal rates and complicated medical devices.*

A quote from one blogger describes how humor can be a source of emotional support:

*It’s really, really hard to allow ourselves to be vulnerable with each other but I find that there is a certain empowerment that comes with allowing ourselves to let go of a little fear. Add in humor, and you’ve hit my sweet spot of emotional support.*

In summary, PWT1D are looking for emotional support and ways to feel understood. Each person has different needs, but as one person commented, “I’d be lost without my DOC”. Not everyone is interested in on-line activities; some want social settings, and some want presentations. One participant advised:

*It’s okay to move on and find a better fit—or start your ideal group yourself. Poke around online until you find the connections you are looking for. We all need different things, and over time the things we need often change and grow.*

### Theme 2: Blogs Were Used as a Source of Sharing Lived User Experience of Having Diabetes, More Often Than as a Source of Communicating Medical Knowledge or Facts About Diabetes

Blogs were a platform to communicate shared lived experience, typically not related to improving HbA1c or discussing complications. Blogs were not often used to seek or offer medical advice, such as reducing HbA1c, complications, or hyper/hypo-glycemia. One area discussed frequently was advances in technology, which were helpful in managing diabetes, but practically could be described as problematic. Many people in the DOC talked about how wearing diabetes technology impacted their lives and made them feel different. By wearing technology on the body, diabetes became more easily visible. It made the awareness of diabetes ever more present by being attached constantly to a device that is keeping the PWT1D alive. For some, the impact of wearing technology was a reason they delayed going on an insulin pump for the inconvenience of “having something attached to me all the time”.

*I think I also wish I’d known that a) seeing the pump for the first time can be surprisingly upsetting (as we discussed, I think it had to do with having a physical reminder of the fact that I had a disease) and b) that I’d get over it. These days, I cannot imagine NOT having my pump. It’s only when I see other people’s reactions to it (Wait, you have to wear that even when you’re sleeping)? that I remember that it’s kind of weird to have to be attached to a machine 24/7 in order to stay alive.*

The benefits of wearing the pump generally outweighed the inconvenience, but it did take time getting used to wearing a device and responding to unsolicited questions. For many PWT1D, wearing a device meant that they received more attention (wanted and unwanted) from strangers, asking about their pumps or continuous glucose monitors (CGMs). This exposure could go either way, with PWT1D becoming more “open and comfortable” about their diabetes at times and for some, but other times and/or for other people, becoming “embarrassed” by having to wear a device.

*While I don’t think there has been a huge emotional impact on me directly related to wearing my pump, I would say that one thing I didn’t consider was that I was making my invisible disease visible. I get a lot more questions about diabetes while wearing a pump than I do when I’m not wearing it or when it is not visible. I don’t mind this though, as it is an opportunity to dispel myths and address people’s curiosity.*

*I lifted the beach blanket by its corners so it would spread out nice and flat. “It is an insulin pump”, I said to them, waving, unaware until that moment how obvious my insulin pump was, clipped to the bottom of my bathing suit, the tubing tucked in kind of haphazardly.*

Another lived experience described was the impact of the alarms that come with pump and/or CGM user. For example, the alarms could draw unwanted attention to the pump or CGM user, such as “Mama? Your [CGM] has beeps!” Additionally, alarms often interrupted coveted sleep. Those without diabetes—the *“other”*—are able to sleep throughout the night without alarms, but those who have alarms lose sleep over them. This was an “insider” view that other people who did not wear technology could not fully understand:

*I was exhausted. I muffled the [CGM] under my pillow so the kid wouldn’t wake up.*

*Can we count the dreams/nightmares about D in that number, too? I’ve taken my CGM off (for about 4 wks now) just to catch up on sleep lost from all the beep beep beeping.*

Although wearing a pump or CGM generated curiosity or attention from strangers, the familiar sound of a device from another person helped form an “instant connection when I see someone on the subway with their tubing sticking out”, as one blogger wrote. She continued, “I know we have something intimate and intense in common”.

There is also the physical impact of wearing a pump, where the “weight and bulk” of the device *can be inconvenient:*

*I’m not sure the emotional side of pump-wearing is different from any method of tight control. However, I do hate the weight & bulk, constantly shifting it according to an activity, and catching loose tubing on doorknobs, etc. OK, on second thought, there IS more stress. If it didn’t provide more flexibility adjusting insulin to activity & better control in conjunction with CGM (also another stressor), I would return to the pen in a heartbeat.*

*The frustration of having to plan out your wardrobe for the week based off where your infusion set is.*

*The size of this stupid transmitter irritates me on a weekly basis at least! Why does it always have to be two steps forward, one step back? Ugh. I would almost rather carry around a larger device just to have a smaller thing on my person. I don’t really NEED any more lumps. Just sayin’.*

Not everyone in the DOC sample of bloggers and commenters was a CGM/pump user and would not be able to comment from the role of an “insider”. One responded:

*I also wish that the community, as a whole, wouldn’t assign pumps as “necessary” for diabetes control. Pumps are a tool that I’m grateful we have available to us, but not using an insulin pump doesn’t equal out to “not trying hard enough”. Injections work really well for some people. Your diabetes may vary.*

Practical informational support came from lived experience that supplemented advice received in a medical office. There were accounts of how people solved problems in everyday life related to diabetes:

*And this is where a reader came in with a suggestion that saved my skin. She wrote, “You need to spray steroid nasal spray on the site after the alcohol or IV prep and before you insert”. She also attached a photo of a rash she received from a CGM and it looked just like mine.*

*I travel frequently and always go through the metal detector with pump in one pocket, [CGM] receiver in another, and transmitter in my abdomen.*

After giving a disclaimer that one should seek medical advice, one commenter commiserated by talking about how he/she used a complicated formula to take insulin for sushi, which was a *“problem food”* for them. There was a comment about someone who put his/her CGM in a glass at night so they could avoid sleeping through the alarm. PWT1D talked about how to get through insurance hassles and how to override denial claims. Others talked about how they bolused or changed their insulin for exercise, but were quick to say that this was their personal experience with a dosing strategy, and it may not work for everyone.

Participants also shared about developments of new clinical trials and their participation in them. This sparked discussion in the comments about various ongoing clinical trials and also brought hope to people.

## Discussion

The primary finding of this study is that T1D blogs provide an exchange of emotional support in dealing with the heavy, unrelenting workload of living with and managing T1D. Blogs empower PWT1D to help each other and PWT1D are able to learn from the people who “get it”.

It is interesting that the blogs did not mention much about how to improve HbA1c or prevent complications of diabetes. One possible reason for this is that these topics are discussed during visits with HCPs, and PWT1D can ask for clinical advice during those visits, leaving less need to pursue such topics with online peers. PWT1D peer support is often emotional in focus, and HCPs may often not be able to easily provide this, even if they want to, because they do not typically have access to the lived experience of having and managing diabetes. HCPs also may have less opportunity to focus on emotional support during appointments that are usually more clinical. Even when PWT1D peer support is informational, it is about lived experience, i.e. “tips and tricks” for managing the diabetes treatment regimen (44), rather than medical information about treatment procedures or efficacy.

There may be some lessons for the healthcare community based on the value that blog-reading PWT1D seem to place on peer support, especially in light of how some note that their peers understand and “get it” better than their HCPs generally do. PWT1D may appreciate if this were simply acknowledged (e.g., that I, as your HCP may not fully understand your day-to-day efforts as well as a peer). They might also appreciate an HCP’s recommendation about where to find online peer support that is trustworthy, as well as sites that might be better to avoid. Even if HCPs cannot provide the same type of support as their peers, HCPs can help them navigate the world of online peer support.

The findings of this study are supported by other research, both using social media and conventional data. Consider the topic of wearable technology. Prior to a recent publication (45), there was little published research that addressed the intrusiveness of wearing visible technology. Whereas that study was conducted *via* survey, the finding of similar results in the current naturalistic blog study of information obtained without prompts strengthens the finding itself, as well as the use of blogs as a source of data. More recently, another blog-based study found that there is a growing trend toward being “out and proud”, including the #showmeyourpump hashtag (44). This is a testimony to the impact of social media in creating culture, specifically a culture that empowers and normalizes PWT1D. In addition, it indicates that monitoring of blog content allows researchers to stay abreast of rapidly developing culture around living with diabetes.

### Strengths and Limitations

One strength of using blogs as a data source is that it is the ultimate naturalistic inquiry; it focuses on topics that are of importance to the participant, and it is unobtrusive in that the research does not influence the nature of the data. Moreover, there is no recall bias. There are no geographic boundaries in that persons can participate in the blog no matter where they live. However, a digital divide may exist in that some people (e.g. people who are older, people who are less educated) are less likely to access the Internet. Also, it is more likely that blog contributors are motivated people with diabetes; thus our results cannot be generalized to PWT1D who do not blog. In addition, it appeared as though many of the PWT1D bloggers were technologically familiar with insulin pumps and CGMs, which is not generalizable to low-end users of technology. We are able to capture data only from bloggers and commenters, and cannot know the experience among “lurkers” (participants who read the blogs but do not actively comment) by analyzing blogs and comments alone; doing so would require interviewing and/or surveying them (12). Further research could explore the experience of lurkers through individual interviews, but that was not the purpose of this study. Also, it was not possible to have specific inclusion/exclusion criteria, or even to know the demographics, much less the identity, of participants; thus, it is not possible to examine how different types of people participate differently in blogging.

The initial research was conducted in 2014. To ensure relevance, we conducted additional analysis in 2019. However, many blogs had stopped activity and some may have moved to other engaging online spaces such as Instagram.

### Conclusion

This study represents a significant reversal of the usual paradigm of discovery and innovation by taking a belief held by a community of PWT1D (that blogs provide them with much-needed support), investigating it, and then bringing it to the healthcare community. More often, concepts come from within the scientific community, are disseminated to the broader clinical community, and then are eventually brought to PWT1D. Our research methodology contributes to the current movement in healthcare to become more patient-centered, in recognition of the primary role people have in managing their chronic conditions. In fact, blogs themselves may facilitate the empowerment and activation of PWT1D from passive to active participant, and from individual to community member (44).

Although not implemented in this paper, another possible use of diabetes blog data is to examine self-management barriers and facilitators. Blogs provide a unique window into patient-driven—rather than HCP-driven—concerns. As such, blogs may offer distinct advantages and economies not only for participants, but also for researchers and the healthcare system. The flexibility in timeliness (not just at scheduled appointment or group meeting times), convenience, and the ability of blogs to reach many individuals at the same time may improve outcomes and reduce costs. Researchers may also save time and money by using existing data sources as a foundation. These dual uses of blog data suggest that blogs are a growing part of health research and care.

## Data Availability Statement

The data presented in the study are included in the article; further inquiries can be directed to the corresponding author.

## Ethics Statement

The Internet ethnography (“netnography”) (41) methods used in this study were approved by the Penn State College of Medicine Institutional Review Board. Identified blog authors gave permission to retrospectively analyze data publicly available on their blogs posted between June 1, 2012 and July 31, 2014.

## Author Contributions

HS was responsible for the draft of the first manuscript, ensuring the codebook, and inter-rater reliability was completed accurately and rigorously. SO reviewed and edited the drafts, discussed the coding and coding procedures, and provided clinical support for unhelpful or unsafe practices mentioned in the blogs. EM is the project manager for the study, whose role includes all administrative tasks as well as being the primary coder for the “first phase” of the data, and reviewed the manuscript for accuracy of the methodology. TO reviewed and edited the drafts, was the secondary coder of the data and ensured rigor of the data, and also provided clinical support. MFP contributed to the design of the stratified, clustered proportionate probability sample, and provided input into the organization of this manuscript. AS coded and analyzed the data from the “second phase” of the blogs and reviewed the final manuscript. All authors contributed to the article and approved the submitted version.

## Funding

Research reported in this publication was supported by the National Institute of Diabetes and Digestive and Kidney Diseases of the National Institutes of Health under Award Number DP3DK104054. The content is solely the responsibility of the authors and does not necessarily represent the official views of the National Institutes of Health.

## Conflict of Interest

The authors declare that the research was conducted in the absence of any commercial or financial relationships that could be construed as a potential conflict of interest. 

The reviewer MMF declared a past co-authorship with several of the authors MP, HS to the handling editor.

## References

[1] Coffen RD. The 600-step program for type 1 diabetes self-management in youth: the magnitude of the self-management task. Postgrad Med (2009) 121(5):119–39. doi: 10.3810/pgm.2009.09.2059 19820281

[2] Greenhalgh T Collard A Campbell-Richards D Vijayaraghavan S Malik F Morris J. Storylines of self-management: narratives of people with diabetes from a multiethnic inner city population. J Health Serv Res Policy (2011) 16(1):37–43. doi: 10.1258/jhsrp.2010.009160 20819914

[3] Hempler NF Joensen LE Willaing I. Relationship between social network, social support and health behaviour in people with type 1 and type 2 diabetes: cross-sectional studies. BMC Public Health (2016) 16(1):198. doi: 10.1186/s12889-016-2819-1 26926867 PMC4772283

[4] Hill K Ward P Gleadle J. “I kind of gave up on it after a while, became too hard, closed my eyes, didn’t want to know about it”—adults with type 1 diabetes mellitus describe defeat in the context of low social support. Heal Expect (2019) 22(2):254–61. doi: 10.1111/hex.12850 PMC6433328 30565796

[5] Lee AA Piette JD Heisler M Janevic MR Rosland A-M. Diabetes self-management and glycemic control: The role of autonomy support from informal health supporters. Heal Psychol (2019) 38(2):122. doi: 10.1037/hea0000710 PMC6442463 30652911

[6] Nicolucci A Kovacs Burns K Holt RIG Comaschi M Hermanns N Ishii H. Diabetes Attitudes, Wishes and Needs second study (DAWN2^TM^): Cross-national benchmarking of diabetes-related psychosocial outcomes for people with diabetes. Diabetes Med (2013) 30(7):767–77. doi: 10.1111/dme.12245 23711019

[7] Stuckey HL Mullan-Jensen CB Reach G Burns KK Piana N Vallis M. Personal accounts of the negative and adaptive psychosocial experiences of people with diabetes in the second Diabetes Attitudes, Wishes and Needs (DAWN2) study. Diabetes Care (2014) 37(9):2466–74. doi: 10.2337/dc13-2536 24973437

[8] Brady E Segar J Sanders C. Accessing support and empowerment online: The experiences of individuals with diabetes. Heal Expect (2017) 20(5):1088–95. doi: 10.1111/hex.12552 PMC5600220 28718928

[9] Oser TK Oser SM McGinley EL Stuckey HL. A novel approach to identifying barriers and facilitators in raising a child with type 1 diabetes: qualitative analysis of caregiver blogs. JMIR Diabetes (2017) 2(2):e27. doi: 10.2196/diabetes.8966 30291073 PMC6238834

[10] Litchman ML Rothwell E Edelman LS. The diabetes online community: older adults supporting self-care through peer health. Patient Educ Couns (2018) 101(3):518–23. doi: 10.1016/j.pec.2017.08.023 28947360

[11] Litchman ML Walker HR Ng AH Wawrzynski SE Oser SM Greenwood DA. State of the science: a scoping review and gap analysis of diabetes online communities. J Diabetes Sci Technol (2019) 13(3):466–92. doi: 10.1177/1932296819831042 PMC6501517 30854884

[12] Oser TK Oser SM Parascando JA Grisolano LA Krishna KB Hale DE. Challenges and Successes in Raising a Child With Type 1 Diabetes and Autism Spectrum Disorder: Mixed Methods Study. J Med Internet Res (2020) 22(6):e17184. doi: 10.2196/17184 32217508 PMC7301267

[13] Oser TK Oser SM Parascando JA Hessler-Jones D Sciamanna CN Sparling K. Social Media in the Diabetes Community: a Novel Way to Assess Psychosocial Needs in People with Diabetes and Their Caregivers. Curr Diabetes Rep (2020) 20(3):1–10. doi: 10.1007/s11892-020-1294-3 32080765

[14] Oser SM Stuckey HL Parascando JA McGinley EL Berg A Oser TK. Glycated hemoglobin differences among blog-reading adults with type 1 diabetes compared with those who do not read blogs: cross-sectional study. JMIR Diabetes (2019) 4(2):e13634. doi: 10.2196/13634 30938693 PMC6465975

[15] Keim-Malpass J Steeves RH Kennedy C. Internet ethnography: A review of methodological considerations for studying online illness blogs. Int J Nurs Stud (2014) 51(12):1686–92. doi: 10.1016/j.ijnurstu.2014.06.003 24985757

[16] Hilliard EM Sparling MK Hitchcock J Oser KT Hood KK. The emerging diabetes online community. Curr Diabetes Rev (2015) 11(4):261–72. doi: 10.2174/1573399811666150421123448 PMC4586085 25901500

[17] Vygotsky LS. Mind in society: The development of higher psychological processes. London, England: Harvard University Press (1980). doi: 10.2307/j.ctvjf9vz4

[18] Dolan P. Patients want to use social media tools to manage health care. Am Med News (2012) 55(18). Available at: https://carlofavaretti.wordpress.com/2012/05/06/patients-want-to-use-social-media-tools-to-manage-health-care/.

[19] Cooper A Kar P. A new dawn: the role of social media in diabetes education. J Diabetes Nurs (2014) 18(2):68–71.

[20] Farrell H. Impact and evidence of social media use amongst people with diabetes. Nursing in General Practice (2014).

[21] Greene JA Choudhry NK Kilabuk E Shrank WH. Online social networking by patients with diabetes: a qualitative evaluation of communication with Facebook. J Gen Intern Med (2011) 26(3):287–92. doi: 10.1007/s11606-010-1526-3 PMC3043192 20945113

[22] Shaffer-Hudkins E Johnson N Melton S Wingert A. Social media use among individuals with diabetes. Int J Commun Heal (2014) 4:38–43. doi: 10.2196/diabetes.8603

[23] Saric M. Blogging statistics and trends you should know in 2020 [Internet]. (2020). Available at: https://howtomakemyblog.com/blogging-statistics/#22-lessspan-classdisplay-inline-block-margin-bottom-7greaterWhat-percentage-of-internet-users-read-blogs-regularly-47lessspangreater.

[24] Sarasohn-Kahn J. The wisdom of patients: Health care meets online social media. Oakland, CA: California HealthCare Foundation (2008).

[25] Chaffey D. Global social media research summary 2020 [Internet]. (2020). Available at: https://www.smartinsights.com/social-media-marketing/social-media-strategy/new-global-social-media-research/.

[26] McMahon KL. Power and pitfalls of social media in diabetes care. Diabetes Spectr (2013) 26(4):232–5. doi: 10.2337/diaspect.26.4.232

[27] Das A Faxvaag A. What influences patient participation in an online forum for weight loss surgery? A qualitative case study. Interact J Med Res (2014) 3(1):e4. doi: 10.2196/ijmr.2847 24509408 PMC3936279

[28] Bair AL. Blogging for dummies. NJ, USA: John Wiley & Sons (2019).

[29] JDFR. Type 1 Diabetes Facts [Internet]. (2020). Available at: https://www.jdrf.org/t1d-resources/about/facts/.

[30] Antheunis ML Tates K Nieboer TE. Patients’ and health professionals’ use of social media in health care: motives, barriers and expectations. Patient Educ Couns (2013) 92(3):426–31. doi: 10.1016/j.pec.2013.06.020 23899831

[31] Patel R Chang T Greysen SR Chopra V. Social media use in chronic disease: a systematic review and novel taxonomy. Am J Med (2015) 128(12):1335–50. doi: 10.1016/j.amjmed.2015.06.015 26159633

[32] Magnezi R Bergman YS Grosberg D. Online activity and participation in treatment affects the perceived efficacy of social health networks among patients with chronic illness. J Med Internet Res (2014) 16(1):e12. doi: 10.2196/jmir.2630 24413148 PMC3906665

[33] Rinker J Dickinson JK Litchman ML Williams AS Kolb LE Cox C. The 2017 diabetes educator and the diabetes self-management education national practice survey. Diabetes Educ (2018) 44(3):260–8. doi: 10.1177/0145721718765446 29589821

[34] Warshaw H Hodgson L Heyman M Oser TK Walker HR Deroze P. The Role and Value of Ongoing and Peer Support in Diabetes Care and Education. Diabetes Educ (2019) 45(6):569–79. doi: 10.1177/0145721719882007 31617467

[35] Stuckey HL Vallis M Burns KK Mullan-Jensen CB Reading JM Kalra S. “I do my best to listen to patients”: qualitative insights into DAWN2 (diabetes psychosocial care from the perspective of health care professionals in the second diabetes attitudes, wishes and needs study). Clin Ther (2015) 37(9):1986–98. doi: 10.1016/j.clinthera.2015.06.010 26169765

[36] Peyrot M Rubin RR Siminerio LM. Physician and nurse use of psychosocial strategies in diabetes care: results of the cross-national Diabetes Attitudes, Wishes and Needs (DAWN) study. Diabetes Care (2006) 29(6):1256–62. doi: 10.2337/dc05-2444 16732005

[37] Snow R Humphrey C Sandall J. What happens when patients know more than their doctors? Experiences of health interactions after diabetes patient education: a qualitative patient-led study. BMJ Open (2013) 3(11):4. doi: 10.1136/bmjopen-2013-003583 PMC3831109 24231459

[38] Aziz Z Riddell MA Absetz P Brand M Oldenburg B Investigators AP for PDP. Peer support to improve diabetes care: an implementation evaluation of the Australasian Peers for Progress Diabetes Program. BMC Public Health (2018) 18(1):262. doi: 10.1186/s12889-018-5148-8 29454327 PMC5816559

[39] Piette JD Resnicow K Choi H Heisler M. A diabetes peer support intervention that improved glycemic control: mediators and moderators of intervention effectiveness. Chronic Illn (2013) 9(4):258–67. doi: 10.1177/1742395313476522 PMC3830685 23585636

[40] Riddell MA Renwick C Wolfe R Colgan S Dunbar J Hagger V. Cluster randomized controlled trial of a peer support program for people with diabetes: study protocol for the Australasian peers for progress study. BMC Public Health (2012) 12(1):843. doi: 10.1186/1471-2458-12-843 23035666 PMC3519788

[41] Kozinets RV. On netnography: Initial reflections on consumer research investigations of cyberculture. ACR North Am Adv (1998) 25:366–71.

[42] Johnson TP. Snowball sampling: introduction. IL, USA: Wiley StatsRef Stat Ref Online (2014). doi: 10.1002/9781118445112.stat05720

[43] Chowdhury MF. Coding, sorting and sifting of qualitative data analysis: Debates and discussion. Qual Quant (2015) 49(3):1135–43. doi: 10.1007/s11135-014-0039-2

[44] Tenderich A Tenderich B Barton T Richards SE. What are PWDs (people with diabetes) doing online? A netnographic analysis. J Diabetes Sci Technol (2019) 13(2):187–97. doi: 10.1177/1932296818813192 PMC6399800 30477335

[45] Hood M Wilson R Corsica J Bradley L Chirinos D Vivo A. What do we know about mobile applications for diabetes self-management? A review of reviews. J Behav Med (2016) 39(6):981–94. doi: 10.1007/s10865-016-9765-3 27412774

